# Association Analysis of LEP Signaling Pathway with Type 2 Diabetes Mellitus in Chinese Han Population from South China

**DOI:** 10.1155/2021/5517364

**Published:** 2021-09-20

**Authors:** Haibing Yu, Lin Xu, Hao Liu, Jialu Huang, Ling Luo, Rong Chen, Jian Xu, Chunwen Lin, Weiying Chen, Yuxun Xie, Huihuang Yang, Danli Kong, Yuanlin Ding

**Affiliations:** School of Public Health, Guangdong Medical University, Dongguan 523808, China

## Abstract

**Objective:**

This study is aimed at analyzing the relationship between leptin (LEP) signaling pathway and type 2 diabetes mellitus (T2DM) and at providing support for molecular genetic research on the pathogenesis of T2DM in Chinese Han population.

**Methods:**

A case-control study was designed, including 1092 cases with T2DM and 1092 healthy controls of Chinese Han origin recruited from ten hospitals in Guangdong Province, Southern China. Twenty-three single nucleotide polymorphisms (SNPs) of 15 genes in LEP signaling pathway were genotyped by SNPscan™ kit. The Pearson chi-square test, Cochran-Armitage trend test, MAX3, and logistic regression were applied to analyze the association between single nucleotide polymorphism (SNP) and T2DM; unconditional logistic regression was used to analyze haplotype in LD block; and SNP set analysis based on logistic kernel machine regression was used to analyze pathway. All statistical analysis was performed by SPSS25.0, R2.14, Haploview4.2, SNPStats, and other statistical software packages.

**Results:**

In association analysis based on SNP, rs2167270 had statistical significance both in the adjusted and unadjusted covariate dominant model and in the unadjusted covariate overdominant model while it had no significant difference in the adjusted covariate overdominant model. Compared to GG genotype, rs2167270 of AG genotype had statistical significance in both the adjusted and unadjusted covariate codominant models. And rs16147 had statistical significance in robust test, stealth model and overdominant model, and adjusting and unadjusting covariate. This study found linkage disequilibrium existed between rs2167270 and rs4731426 of LEP, rs10889502 and rs17127107 of JAK1, rs2970847 and rs6821591 of PPARGC1A, rs249429 and rs3805486 of PRKAA1, rs1342382 and rs6588640 of PRKAA2, rs3766522 and rs6937 of PRKAB2, rs2970847 and rs6821591 of PRKAG2, and rs6436094 and rs645163 of PRKAG3. There was no positive finding with statistical significance from the unconditional logistic regression of the mentioned genes' haplotype of LD block.

**Conclusions:**

LEP signaling pathway association with T2DM remained to be confirmed in Chinese Han population, although rs2167270 and rs16147 were significantly associated with T2DM.

## 1. Introduction

Diabetes has become a major public problem around the world [1], with the numbers increasing from 108 million in 1980 to 422 million in 2014 [2], and the number of people with diabetes is expected to increase to 592 million by 2035 [3]. Type 2 diabetes mellitus (T2DM) is the most prevalent form of diabetes mellitus (DM), accounting for about 90-95% of the total number of DM cases worldwide [4].

Insulin resistance is the basic pathophysiological change of type 2 diabetes, almost throughout the entire process of the occurrence and development of type 2 diabetes [5]. Studies have shown that free fatty acid (FFA) and adipocytokines released by adipose tissue can cause insulin resistance through a variety of ways [6–9].

For a long time, it has been believed that adipose tissue is only involved in the body's energy storage and nontrembling thermogenesis. With the discovery of leptin (LEP) in 1994 [10], a series of new functions of adipose tissue were revealed. Adipose tissue is not only an energy storage organ of the body but also an endocrine organ [11]. It is known that human adipocytes secrete dozens of adipocytokines and protein factors, such as leptin, adiponectin, resistin, and visfatin. Some of these adipocytokines enter the human blood circulation to regulate the functions of distant target organs, and the other part plays a role of adjacent secretion or autocrine regulation in adjacent tissues or cells or within cells.

Leptin is a secreted protein synthesized by fat cells. It is the coded product of obese gene (ob gene). It exists freely in the blood or binds to leptin-binding protein. It has a wide range of centers such as reducing food intake and increasing energy consumption. And peripheral tissue effects are mediated through leptin receptors [12]. Leptin receptors belong to the class I cytokine receptor family and are widely distributed in central and peripheral tissues. Recent research results have shown that the occurrence and development of obesity, insulin resistance, and type 2 diabetes are closely related to the abnormal function of adipocytokines or their receptors [13–15]. Leptin can cause insulin resistance and hyperinsulinemia through a variety of ways, and hyperinsulinemia and insulin resistance can cause excessive secretion of leptin, thus forming a vicious circle, leading to abnormal glucose and lipid metabolism [12, 16].

Single nucleotide polymorphism mainly refers to the polymorphism of DNA sequence caused by the variation of single nucleotide at the genome level. It is the most common type of human heritable variation. Recent studies have shown that there is an association between SNPs in multiple genes and T2DM and its complications. Letonja et al.'s [17] group finds a significant association between the SIRT1 rs7069102 polymorphism and diabetic nephropathy caused by T2DM. Zhang et al.'s [18] group discovers that rs62099905 in the CNDP1 gene was related to the serum glucose level of healthy Chinese Han population. Cui et al.'s [19] study shows that ADIPOQ gene polymorphisms rs10937273, rs1501299, rs182052, rs2241767, and rs266729 are associated with type 2 diabetes. Zhu et al.'s [20] study suggests that the interaction effects of SNP-SNP and SNP-environmental factors were related to T2DM susceptibility, and Khan et al.'s [21] research shows that the genetic variation of the TCF7L2 gene is related to the increased susceptibility to T2DM. Juttada et al.'s [22] group discovers that TCF7L2 gene polymorphism is closely related to a positive family history of diabetes. Bains et al.'s [23] study suggests that LEPrs7799039 and LEPRrs1137101 polymorphism conferred 3.4- and 2.1-fold risk towards the development of T2DM after adjustment of various covariates under a recessive genetic model.

This study intends to analyze the association between a SNP and type 2 diabetes based on the LEP signaling pathway, hoping to discover new mutation sites that may cause insulin resistance, conduct linkage disequilibrium analysis and haplotype-based association analysis, and analyze multiple loci of a single gene. Evaluate the distribution difference of haplotypes formed by points between the case group and the control group; conduct SNP-SNP interaction analysis to find meaningful interaction pairs; use pathway analysis to explore the relationship between LEP signaling pathway and T2DM.

## 2. Materials and Methods

### 2.1. Study Population

During November 2011 to October 2013, case-control groups of 1092 patients diagnosed as having T2DM were recruited from 10 hospitals in Zhanjiang, Maoming, Shaoguan, Dongguan, and Shenzhen of Guangdong Province. T2DM was diagnosed according to the criteria of the World Health Organization (WHO) in 1999. The inclusive criteria of the T2DM patient group were as follows: (1) range of ages 20-70; (2) random blood glucose levels ≥ 11.1 mmol/l with diabetes symptoms including polydipsia, polyphagia, polyuria, weight loss, itchiness, blurred vision, and other acute metabolic disorders caused by hyperglycemia and fasting plasma glucose levels ≥ 7.0 mmol/l without diabetes symptoms or blood glucose levels ≥ 11.1 mmol/l with a glucose tolerance test after two hours oral dose; and (3) patients without other serious diseases, such as cardiovascular and cerebrovascular diseases, malignant tumors, and other diseases. The control group comprised 1092 normal persons who were examined in the same hospital at the same time as the cases. The inclusive criteria of the control group were as follows: (1) range of ages 20-70; (2) without a family history of diabetes; and (3) healthy after physical examination including medical history, blood glucose, and other biochemical test results. This study was approved by the Ethics Committee. All the surveys and samples obtained the consents of participants in advance, and the informed consent forms had been legally consented. All the participants were the permanent residents of Chinese Han nationality in Guangdong Province, and there is no blood relationship among them.

### 2.2. Information Collection and Blood Sample Collection

A standardized survey is aimed at respondents who met the inclusion criteria, including age, gender, place of origin, occupation, disease history, duration of illness, smoking history, family history, complications, and diet, and exercise was conducted by a uniformly trained investigator. At the end of the investigation, the height and weight of the subjects were measured by a height and weight scale, and the blood pressure of the right arm in a sitting position was measured with a benchtop mercury sphygmomanometer, twice, and averaged.

5 ml of peripheral blood was collected by the endocrine nurses in the morning, and the relative clinical biochemical indicators, including fasting plasma glucose (FPG), total cholesterol (TC), triglyceride (TG), high-density lipoprotein cholesterol (HDL-C), low-density lipoprotein cholesterol (LDL-C), and glycosylated hemoglobin A1c (HbA1c), were detected. The FPG level was measured by the glycosylation method, and the plasma levels of TC, TG, HDL-C, and LDL-C were determined in an enzymatic method. HBA1C was determined by a high-performance liquid chromatography (BIO-RADD-10TM glycated hemoglobin detection system), and other biochemical indicators were detected by an automatic biochemical analyzer. Besides, 4 ml of peripheral blood of the subjects (2 ml per tube) was collected, anticoagulated with EDTA·k2, and stored at -80°C.

This study was approved by the ethics committee, and all the investigations and sampling were subject to the consent of the subjects with signed informed consent.

### 2.3. Data Collation and Database Establishment

All completed questionnaires were uniformly coded, and all participants' questionnaire information, physical examination, and clinical biochemical examination results were compiled. We used EpiData3.1 software to build a database with data by double input. The entered data was checked by both manual and computer methods to ensure that the data has no logic errors and no entry errors.

### 2.4. DNA Extraction

1. Transfer the blood sample from the anticoagulation tube to a 10 ml centrifuge tube, and mark the centrifuge tube
2. Add 2 ml of cell lysate CL to blood containing anticoagulant, mix upside down, centrifuge at 5000 rpm for 10 min, and discard the supernatant
3. Add 3 ml of cell lysate CL to it, mix upside down, centrifuge at 5000 rpm for 10 min, discard the supernatant, and put the centrifuge tube upside down on clean absorbent paper for 1 min and dry by airing
4. Configure a mixture of buffer FG and proteinase K (1 ml buffer + 10 *μ*l proteinase K, ready for use)
5. Add 1 ml of mixed solution to each sample and vortex immediately until the solution is free of clumps
6. Perform water bath at 65°C for 20 minutes, slowly tilting and inverting and mixing several times during this period
7. Add 1 ml of isopropanol, and mix thoroughly by inversion until filaments or clusters (DNA) appear
8. Centrifuge at 5000 rpm for 15 minutes, discard the supernatant, and place the centrifuge tube upside down on a clean absorbent paper to ensure that the precipitate is in the tube
9. Add 1 ml of 70% ethanol, transfer to a 1.5 ml centrifuge tube, centrifuge at 8000 rpm for 10 min in a small centrifuge, and discard the supernatant
10. Add 1 ml of 70% ethanol, centrifuge at 8000 rpm for 10 min in a small centrifuge, and discard the supernatant
11. Dry the DNA pellet in the centrifuge tube at room temperature until the liquid evaporates
12. Add the corresponding amount of buffer TB according to the amount of DNA; heat at 65°C for 1 hour to dissolve the DNA, during which time the finger flicks several times to aid the solution; and finally centrifuge for 30 s to shake the undissolved precipitate to the bottom of the tube
13. Perform DNA quality detection with a nucleic acid protein content detector, record DNA concentration (ng/*μ*l) and purity (OD260/280 value, OD260/230 value), and finally store the DNA in a refrigerator at -80°C

### 2.5. Screening and Typing of SNP

The pathway diagram of LEP signaling pathway was obtained from the KEGG database (Kyoto Encyclopedia of Genes and Genomes), and 15 main genes were selected, including LEP, LEPR, JAK (JAK1, JAK2), STAT3, SHP-2 (PTPN11), PPARGC1Alpha (PPARGC1A), AMPK (PRKAA1, PRKAA2, PRKAB2, PRKAG1, PRKAG2, and PRKAG3), alpha-MSH (POMC), and NPY. Haploview (ver.4.2) was used to select tagSNPs from selected genes and their upstream and downstream 5 kb regions, requiring MAF (minor allele frequency) > 0.05 and *r*^2^ ≥ 0.8 (SNP MAF and LD data come from the HapMap database, release 27, http://snp.cshl.org/). Then, use FastSNP (http://fastsnp.ibms.sinica.edu.tw/pages/input_CandidateGeneSearch.jsp) to predict the potential function of tagSNP, select tagSNP with higher prediction score, and select 1-2 tagSNP for each gene. Finally, 23 SNPs were selected from 15 genes. These SNPs were distributed on 8 chromosomes, one of which was located downstream of the 3′ end, 4 of which were located in the untranslated region of the 3′ end, 2 of which were located upstream of the 5′ end, one of which was located in the 5′untranslated region, 13 of which were located in the intron region, and 2 of which were located in the exon region. In the Chinese population, the minimum MAF was 0.128 (rs3766522) and the maximum MAF was 0.444 (rs6436094). Related information is listed in Table 1.

### 2.6. Statistical Analysis

Comparisons of all variables between T2DM cases and control subjects were carried out by the *χ*^2^ test for categorical variables or *t*-test for continuous variables. The Pearson chi-square test, Cochran-Armitage trend test, MAX3, and logistic regression were used to analyze the association between SNP and T2DM; unconditional logistic regression was used to analyze haplotype in LD block; and SNP set analysis based on logistic kernel machine regression was used to analyze pathway. All statistical analysis was performed by SPSS25.0, PLINK 1.07, R 2.14.2, Haploview 4.2, SNPStats, and other statistical software packages.

## 3. Results

### 3.1. Equilibrium Test Results of Baseline Data in the Case Group and Control Group

In this study, 1092 cases and 1092 controls were included, and the individuals with the loss rate of SNP type > 20% were excluded. Eventually, 1067 cases and 1054 controls were included in the following analysis, including 532 males and 535 females in the case group with an average age of 49.86 years and 532 males and 522 females in the control group with an average age of 50.47 years. Differences of the age composition, body mass index (BMI), fasting plasma glucose (FPG), triglyceride level, and low-density cholesterol level between the two groups were statistically significant. The average age, BMI, FPG, and triglyceride level of the case group are higher than those of the control group, as shown in Table 2.

### 3.2. SNP Typing Results

The typing success rate of 23 SNPs was all above 98%, and the minimum allele frequency was 0.119, and the maximum was 0.474. The Hardy-Weinberg equilibrium test shows that each point satisfies the Hardy-Weinberg equilibrium, as shown in Table 3.

### 3.3. Allele Association Analysis Results

The results of genotype association analysis are shown in Table 4. There was no statistical difference in the suballelic frequency of each SNP between the case group and the control group (*P* > 0.05). After adjusting for covariates such as age and BMI, there was no statistical difference in the suballelic frequency between the case group and the control group.

### 3.4. Genotype Association Analysis Results

The genotype (CC/CT/TT) distribution of rs16147 was statistically significant between the two groups (*χ*^2^ = 7.275, *P* = 0.026). There was no statistical difference in genotype distribution of other SNPs, as shown in Table 5.

In order to further confirm whether each SNP was associated with T2DM and whether the incidence of disease increases with the increase of risk alleles in the genotype, we conducted the Cochran-Armitage trend test under different genetic models (additive model, codominant model, dominant model, invisible model, and superdominant model), and the results are shown in Table 6.

Since the trend test relies on prespecified scores, different scores correspond to different genetic models. Only when the designated gene model is a true gene model the corresponding trend test is the test with the best power. We further applied the robust test method and compared the results obtained with the methods based on various genetic models (see Table 7 for details). The result of rs16147 was statistically significant (*P* = 0.045). The other SNPs were not statistically significant in the robust test results.

After adjusting the covariate, the results under the five genetic models are shown in Table 8. rs2167270 still has statistical significance in the dominant model (*P*_adj_ = 0.039, OR = 1.20, 95% CI: 1.01 1.44), while there is no statistical significance in the superdominant model; in the codominant model, compared to GG genotype, AG genotype (OR = 1.22, 95% CI: 1.01 1.47, *P*_adj_ = 0.024) is statistically significant, and compared to GG genotype, AA genotype (OR = 1.12, 95% CI: 0.78 1.63, *P*_adj_ = 0.028) is still not statistically significant. rs16147 was still statistically significant in both the stealth model and the superdominant model, in the recessive model (*P*_adj_ = 0.012, OR = 0.72, 95% CI: 0.56-0.93), and in the superdominant model (*P*_adj_ = 0.044, OR = 1.21, 95% CI: 1.01-1.44).

### 3.5. Linkage Disequilibrium Analysis and Association Analysis Based on Haplotype

Linkage is a genetic tendency where genetic markers are inherited together as a result of being near to one another on the same chromosome. Genetic linkage analysis, one of the old study approaches, focuses on genomic regions with large genetic effect that can influence the development of a disease [24, 25].

**Figure 1:**
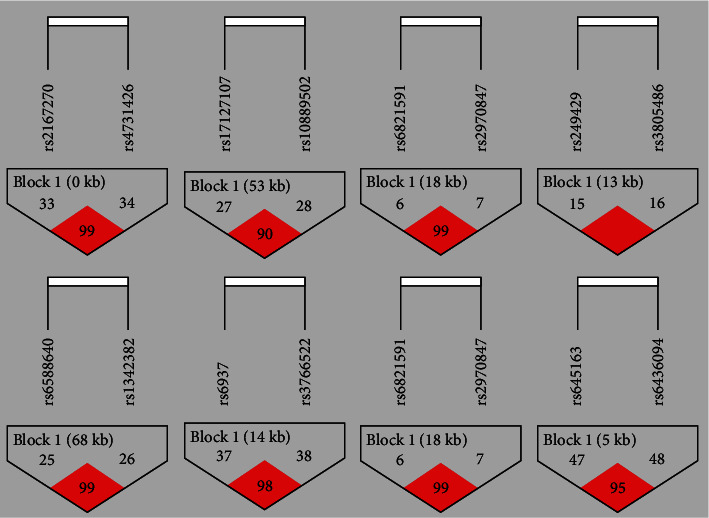
Results of linkage disequilibrium analysis of 8 genes in the LEP signaling pathway.

Haploview 4.2 software was used to analyze the linkage imbalance (LD) between different loci in the same gene, and the results showed that there was linkage imbalance between loci in 8 genes including LEP.  Figure 1 shows the composition of LD block of 8 genes including LEP.

The haplotype in LD block was analyzed by using SNPStats software. The analysis results are shown in Table 9. The haplotype of LD block in each gene was not found to be statistically significant.

### 3.6. SNP-SNP Interaction Results

We analyzed 23 SNPS of 15 genes under the pathways of the LEP pathway by logistic regression model-based SNP-SNP interaction analysis, and the partial results are shown in Table 10. According to the test level of 0.01, 2 pairs of statistically significant results were obtained. The OR value of the interaction term is 1.311, which corresponds to the interaction between rs16147 and rs1044471, and the corresponding genes are NPY and ADIPOR2. The OR value of the interaction term is 1.422, which corresponds to the interaction between rs2167270 and rs5435, and the corresponding genes are LEP and SLC2A4.

**Figure 2:**
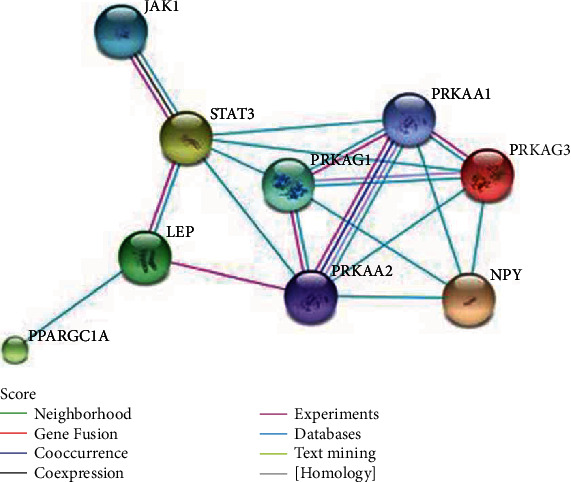
The interaction map of 8 genes in the LEP signaling pathway.

We uploaded 8 genes from the LEP signaling pathway to the STRING (Search Tool for the Retrieval of Interacting Genes/Proteins) tool. The interaction between proteins encoded by these genes was analyzed, and the results are shown in Figure 2.

### 3.7. Pathway Analysis Result

Four kinds of kernel functions linear, linear.weighted, IBS, and IBS.weighted were used to analyze the SNP set based on pathways. The results are shown in Table 11. No statistical significance was found whether covariates were added or not (*P* > 0.05), nor was the empirical *P* value obtained by bootstrap method.

## 4. Discussion

The prevalence of diabetes increases significantly in recent decades, affecting about 6% of adult population globally. Therefore, it is one of the major health care challenges in the world [26]. In China, the incidence of T2D in adults has been increasing over recent decades [27]. T2DM is a major chronic disease that is affected by genetic and environmental factors [28]. The development of T2DM involves multiple factors and signaling pathways, such as hyperglycemia, dyslipidemia, and oxidative damage. The main pathological features are insulin resistance and damage to the function of cells, which is a chronic metabolic disease caused by the interaction of genetic and environmental factors [29, 30].

Heredity has all along been mentioned to play an important role in the development of diabetes [31]. Genome-wide association studies have identified more than 100 independent SNPS associated with T2DM regulation and risk [32]. Several gene polymorphisms of leptin have been reported to be associated with T2DM [2, 33]. For example, the leptin (LEP) G2548A polymorphism has been associated with increased leptin production and plasma secretion from adipocytes [34]. Human LEP is located on chromosome7q31.3, and its translational product is leptin, which plays a decisive role in the regulation of human appetite and results in severe metabolic disorders [35]. Such signalization upregulates and downregulates genes involved in cell differentiation, proliferation, apoptosis, and synthesis of extracellular matrix proteins [36].

In this study, rs2167270 and rs16147 were associated with type 2 diabetes in the unit-point correlation analysis. However, in order to further confirm its association with type 2 diabetes, independent sample verification is needed in Chinese Han population. Linkage disequilibrium analysis and correlation analysis based on single type times get within the eight genes of the pathway between the two respective loci; there is a linkage disequilibrium relationship; each genotype in LD block of haploid analysis has not been having a positive result. The LEP pathway gene SNP-SNP interactions analysis results have statistical significance and illustrate that the SNP-SNP interactions (gene interactions) are part of the genetic structure of type 2 diabetes mellitus. LEP gene SNP in pathway, the statistically significant results in the occurrence of type 2 diabetes, one of more than just a channel or certain genes, plays an important role, may also involve two pathways even based on the joint action of multiple pathways between LEP pathways of SNP set correlation analysis, and has statistically significant results. Considering that a few SNPS were selected in each gene, it is necessary to increase the number of SNPS in follow-up studies.

However, the sample of this study is only the Han population in Guangdong Province. The research group has certain limitations. It is necessary to further verify in other populations to provide new clues for the prevention and treatment strategy of T2DM.

## 5. Conclusion

In summary, we found that rs2167270 and rs16147 may interact to affect the risk of T2DM but remained to further confirm their association, and independent sample validation is required in the Chinese Han population. According to the logistic kernel machine regression results, we cannot think the LEP signaling pathway is associated with T2DM.

## Figures and Tables

**Table 1:** Basic information of 23 tagSNPs selected from 15 genes in the LEP signaling pathway.

Gene	Chr	Position_37	SNP	Region	Allele		MAF
					Minor	Major	
LEP	7	127882070	rs4731426	Intron 1	G	C	0.202
	7	127881349	rs2167270	5′UTR	G	A	0.175
LEPR	1	66063117	rs12405556	Intron 7	G	T	0.153
JAK1	1	65326908	rs17127107	Intron 8	C	G	0.321
	1	65379982	rs10889502	Intron 1	G	C	0.375
JAK2	9	4988761	rs7849191	Intron 2	C	T	0.420
STAT3	17	40507980	rs9891119	Intron 1	A	C	0.357
SHP-2	12	112942353	rs4767860	Intron 14	G	A	0.409
PPARGC1A	4	23815924	rs2970847	Exon 8	T	C	0.204
	4	23797000	rs6821591	3′UTR	C	T	0.292
PRKAA1	5	40782239	rs249429	Intron 1	C	T	0.255
	5	40796045	rs3805486	Intron 1	A	G	0.212
PRKAA2	1	57177388	rs1342382	3′UTR	T	A	0.252
	1	57108423	rs6588640	5′ upstream	A	G	0.168
PRKAB2	1	146626922	rs6937	3′UTR	T	C	0.409
	1	146641686	rs3766522	Intron 2	A	T	0.128
PRKAG1	12	49390677	rs10783299	Exon 2	T	C	0.346
PRKAG2	7	151255203	rs5017427	Intron 15	A	G	0.261
	7	151502302	rs9648724	Intron 1	G	A	0.245
PRKAG3	2	219682257	rs645163	3′ downstream	C	T	0.416
	2	219687597	rs6436094	3′UTR	A	G	0.444
Alpha-MSH	2	25384833	rs6713532	Intron 2	T	C	0.374
NPY	7	24323410	rs16147	5′ upstream	T	C	0.332

**Table 2:** Comparison of baseline data between the case group and control group.

Parameters	T2DM	Control	*P*
*n*	1067	1054	—
Male (%)	532 (49.86)	532 (50.47)	0.080
Age (years)	59.71 ± 11.87	57.23 ± 10.41	<0.001
BMI (kg/m^2^)	24.60 ± 3.24	23.58 ± 3.33	<0.001
FPG (mmol/l)	10.46 ± 4.50	5.60 ± 1.60	<0.001
TC (mmol/l)	5.31 ± 1.59	5.43 ± 1.27	0.056
Triglyceride (mmol/l)	2.24 ± 1.03	1.31 ± 0.96	<0.001
HDL-C (mmol/l)	1.35 ± 0.54	1.37 ± 0.42	0.398
LDL-C (mmol/l)	2.73 ± 1.04	3.03 ± 0.65	<0.001
Hypertension (%)	396 (37.11)	380 (36.05)	0.257
Heart rate	76.40 ± 15.26	76.20 ± 10.92	0.682

BMI (body mass index) = body weight/(height∗height).

**Table 3:** 23SNP genotyping results for 15 genes in the LEP signaling pathway.

Gene	SNP	Allele		Call rate (%)	MAF	*P*_HWE_
		Minor	Major			
LEP	rs4731426	G	C	98.37	0.303	0.161
	rs2167270	A	G	98.34	0.238	0.095
LEPR	rs12405556	G	T	98.34	0.158	1.002
JAK1	rs17127107	C	G	98.32	0.314	0.152
	rs10889502	G	C	98.37	0.340	0.943
JAK2	rs7849191	C	T	98.37	0.369	0.351
STAT3	rs9891119	A	C	98.37	0.445	0.851
SHP-2	rs4767860	G	A	98.24	0.347	0.842
PPARGC1A	rs2970847	T	C	98.37	0.241	0.153
	rs6821591	C	T	98.37	0.328	0.672
PRKAA1	rs249429	C	T	98.37	0.220	0.473
	rs3805486	A	G	98.34	0.234	0.111
PRKAA2	rs1342382	T	A	98.34	0.253	0.332
	rs6588640	A	G	98.37	0.251	0.574
PRKAB2	rs6937	T	C	98.34	0.451	0.421
	rs3766522	A	T	98.37	0.119	0.642
PRKAG1	rs10783299	T	C	98.34	0.474	0.622
PRKAG2	rs5017427	A	G	98.37	0.290	0.563
	rs9648724	G	A	98.29	0.235	0.353
PRKAG3	rs645163	C	T	98.37	0.422	0.902
	rs6436094	A	G	98.37	0.450	0.803
Alpha-MSH	rs6713532	T	C	98.37	0.411	0.056
NPY	rs16147	T	C	98.24	0.362	0.0051

**Table 4:** Results of association analysis between LEP signaling pathway allele and type2 diabetes.

Gene	SNP	Allele	Nondiabetic controls			T2DM patients			*P* value		OR (95% CI)	
			A	a	MAF	A	a	MAF	Observed	Adjusted	Observed	Adjusted
LEP	rs4731426	C/G	1488	620	0.294	1460	674	0.316	0.132	0.141	1.11 (0.97-1.26)	1.11 (0.97-1.26)
	rs2167270	G/A	1630	478	0.227	1599	535	0.251	0.071	0.079	1.14 (0.99-1.31)	1.14 (0.99-1.31)
LEPR	rs12405556	T/G	1775	333	0.158	1802	332	0.156	0.833	0.850	0.98 (0.83-1.16)	0.98 (0.83-1.16)
JAK1	rs17127107	C/G	1454	654	0.310	1451	683	0.320	0.491	0.610	1.05 (0.92-1.20)	1.04 (0.91-1.18)
	rs10889502	C/G	1400	708	0.336	1385	749	0.351	0.310	0.292	1.07 (0.94-1.22)	1.07 (0.94-1.22)
JAK2	rs7849191	C/T	1330	778	0.369	1349	785	0.368	0.932	0.963	0.99 (0.88-1.13)	1.00 (0.88-1.13)
STAT3	rs9891119	A/C	1170	938	0.445	1159	975	0.457	0.431	0.451	1.05 (0.93-1.18)	1.05 (0.93-1.19)
SHP-2	rs4767860	A/G	1401	705	0.335	1390	742	0.348	0.360	0.334	1.06 (0.93-1.21)	1.07 (0.94-1.22)
PPARGC1A	rs2970847	C/T	1604	504	0.239	1623	511	0.239	0.982	0.872	1.00 (0.87-1.15)	1.01 (0.88-1.16)
	rs6821591	T/C	1419	689	0.327	1442	692	0.324	0.863	0.782	0.99 (0.87-1.12)	0.98 (0.86-1.12)
PRKAA1	rs249429	T/C	1654	454	0.215	1652	482	0.226	0.411	0.543	1.06 (0.92-1.23)	1.05 (0.90-1.21)
	rs3805486	A/G	1608	500	0.237	1642	492	0.231	0.614	0.961	0.96 (0.84-1.11)	1.00 (0.86-1.15)
PRKAA2	rs1342382	T/A	1567	541	0.257	1598	536	0.251	0.680	0.721	0.97 (0.84-1.12)	0.97 (0.85-1.12)
	rs6588640	G/A	1567	541	0.257	1611	523	0.245	0.392	0.373	0.94 (0.82-1.08)	0.94 (0.81-1.08)
PRKAB2	rs6937	C/T	1167	941	0.446	1131	1002	0.470	0.123	0.272	1.10 (0.97-1.25)	1.07 (0.95-1.22)
	rs3766522	A/T	1875	233	0.111	1864	270	0.127	0.112	0.241	1.17 (0.97-1.41)	1.12 (0.93-1.36)
	rs10783299	T/C	1114	994	0.472	1152	980	0.460	0.451	0.531	0.95 (0.85-1.08)	0.96 (0.85-1.09)
PRKAG2	rs5017427	G/A	1475	633	0.300	1541	593	0.278	0.112	0.130	0.90 (0.79-1.02)	0.90 (0.79-1.03)
	rs9648724	G/A	1605	503	0.239	1621	511	0.240	0.933	0.990	1.01 (0.87-1.16)	1.00 (0.86-1.16)
PRKAG3	rs645163	C/T	1211	897	0.426	1205	929	0.435	0.521	0.654	1.04 (0.92-1.17)	1.03 (0.91-1.16)
	rs6436094	G/A	1175	933	0.443	1196	938	0.440	0.840	0.992	0.99 (0.88-1.11)	1.00 (0.88-1.13)
Alpha-MSH	rs6713532	C/T	1241	867	0.411	1257	877	0.411	0.982	0.922	1.00 (0.88-1.13)	1.01 (0.89-1.14)
NPY	rs16147	C/T	1343	765	0.363	1382	748	0.351	0.432	0.331	0.95 (0.84-1.08)	0.94 (0.83-1.07)

**Table 5:** Comparison of genotype frequencies between the case group and control group in the LEP signaling pathway.

Gene	SNP	Genotype	Nondiabetic controls	T2DM patients	*P* value	
					*χ* ^2^	*P* value
LEP	rs4731426	CC/CG/GG	535/418/101	499/462/106	3.495	0.174
	rs2167270	GG/GA/AA	640/350/64	598/403/66	5.107	0.078
LEPR	rs12405556	TT/TG/GG	747/281/26	763/276/28	0.209	0.901
JAK1	rs17127107	CC/CG/GG	491/472/91	490/471/106	1.065	0.587
	rs10889502	CC/CG/GG	464/472/118	441/503/123	1.594	0.451
JAK2	rs7849191	CC/CT/TT	412/506/136	420509/138	0.021	0.990
STAT3	rs9891119	AA/AC/CC	323/524/207	315/529/223	0.640	0.726
SHP-2	rs4767860	AA/AG/GG	464/473/116	452/486/128	0.844	0.656
PPARGC1A	rs2970847	CC/CT/TT	619/366/69	624/375/68	0.057	0.972
	rs6821591	TT/TC/CC	474/471/109	492/458/117	0.721	0.697
PRKAA1	rs249429	TT/TC/CC	653/348/53	639/374/54	1.018	0.601
	rs3805486	AA/AG/GG	623/362/69	633/376/58	1.218	0.544
PRKAA2	rs1342382	TT/TA/AA	576/415/63	598/402/67	0.663	0.718
	rs6588640	GG/GA/AA	586/395/73	609/393/65	0.832	0.660
PRKAB2	rs6937	CC/CT/TT	316/535/203	285/562/220	2.867	0.238
	rs3766522	AA/AT/TT	832/211/11	812/240/15	2.644	0.267
PRKAG1	rs10783299	TT/TC/CC	290/534/230	308/536/222	0.619	0.734
PRKAG2	rs5017427	GG/GA/AA	520/435/99	561/419/87	2.549	0.280
	rs9648724	GG/GA/AA	605/395/54	610/401/55	0.007	0.996
PRKAG3	rs645163	CC/CT/TT	349/513/192	344/517/206	0.464	0.793
	rs6436094	GG/GA/AA	325/525/204	341/514/212	0.575	0.750
Alpha-MSH	rs6713532	CC/CT/TT	350/541/163	365/527/175	0.845	0.656
NPY	rs16147	CC/CT/TT	449/445/160	442/498/125	7.275	**0.026**

**Table 6:** Association analysis results under five genetic models in the LEP pathway (OR + *P*).

SNP	Additive		Codominant				Dominant		Recessive		Overdominant	
	*P*	OR (95% CI)	*P*		OR (95% CI)		*P*	OR (95% CI)	*P*	OR (95% CI)	*P*	OR (95% CI)
			1	2	1	2						
rs4731426	0.129	1.11 (0.97-1.26)	0.171	0.111	1.19 (0.99-1.42)	1.13 (0.83-1.52)	0.066	1.17 (0.99-1.39)	0.781	1.04 (0.78-1.39)	0.089	1.16 (0.98-1.38)
rs2167270	0.071	1.14 (0.99-1.31)	0.022	0.5933	1.23 (1.03-1.48)	1.10 (0.77-1.58)	0.029	1.21 (1.02-1.44)	0.910	1.02 (0.72-1.45)	0.028	1.22 (1.02-1.46)
rs12405556	0.831	0.98 (0.83-1.16)	0.902	0.871	0.96 (0.79-1.17)	1.05 (0.61-1.82)	0.753	0.97 (0.80-1.17)	0.820	1.07 (0.62-1.83)	0.681	0.96 (0.79-1.16)
rs17127107	0.485	1.05 (0.92-1.20)	0.592	0.361	1.00 (0.84-1.20)	1.17 (0.86-1.59)	0.762	1.03 (0.87-1.22)	0.312	1.17 (0.87-1.57)	0.772	0.97 (0.82-1.16)
rs10889502	0.295	1.07 (0.94-1.22)	0.454	0.391	1.12 (0.94-1.34)	1.10 (0.83-1.46)	0.211	1.12 (0.94-1.33)	0.813	1.03 (0.79-1.35)	0.284	1.10 (0.93-1.30)
rs7849191	0.934	1.00 (0.88-1.13)	0.990	0.952	0.99 (0.82-1.19)	1.00 (0.76-1.31)	0.901	0.99 (0.83-1.18)	0.982	1.00 (0.78-1.29)	0.893	0.99 (0.83-1.17)
rs9891119	0.435	1.05 (0.93-1.19)	0.730	0.573	1.04 (0.85-1.26)	1.10 (0.86-1.41)	0.572	1.05 (0.88-1.27)	0.473	1.08 (0.87-1.34)	0.950	0.99 (0.84-1.18)
rs4767860	0.361	1.06 (0.93-1.21)	0.660	0.591	1.05 (0.88-1.26)	1.13 (0.85-1.50)	0.442	1.07 (0.90-1.27)	0.473	1.10 (0.84-1.44)	0.761	1.03 (0.87-1.22)
rs2970847	0.978	1.00 (0.87-1.15)	0.974	0.910	1.02 (0.85-1.22)	0.98 (0.69-1.39)	0.911	1.01 (0.85-1.20)	0.871	0.97 (0.69-1.37)	0.840	1.02 (0.85-1.22)
rs6821591	0.858	0.99 (0.87-1.12)	0.702	0.690	0.94 (0.78-1.12)	1.03 (0.77-1.38)	0.602	0.96 (0.81-1.13)	0.640	1.07 (0.81-1.41)	0.412	0.93 (0.78-1.11)
rs249429	0.412	1.06 (0.92-1.23)	0.604	0.593	1.10 (0.92-1.32)	1.04 (0.70-1.54)	0.331	1.09 (0.92-1.30)	0.972	1.01 (0.68-1.49)	0.322	1.09 (0.91-1.31)
rs3805486	0.615	0.97 (0.84-1.11)	0.542	0.313	1.02 (0.85-1.23)	0.83 (0.57-1.19)	0.921	0.99 (0.83-1.18)	0.281	0.82 (0.57-1.18)	0.671	1.04 (0.87-1.24)
rs1342382	0.680	0.97 (0.84-1.12)	0.724	0.690	0.93 (0.78-1.12)	1.02 (0.71-1.47)	0.521	0.95 (0.80-1.12)	0.773	1.05 (0.74-1.50)	0.423	0.93 (0.78-1.11)
rs6588640	0.388	0.94 (0.82-1.08)	0.662	0.551	0.96 (0.80-1.15)	0.86 (0.60-1.22)	0.490	0.94 (0.79-1.12)	0.441	0.87 (0.62-1.23)	0.762	0.97 (0.82-1.16)
rs6937	0.122	1.10 (0.97-1.25)	0.240	0.213	1.16 (0.95-1.42)	1.20 (0.94-1.54)	0.095	1.17 (0.97-1.42)	0.433	1.09 (0.88-1.35)	0.380	1.08 (0.91-1.28)
rs3766522	0.105	1.17 (0.97-1.41)	0.272	0.431	1.17 (0.95-1.44)	1.40 (0.64-3.06)	0.121	1.18 (0.96-1.44)	0.450	1.35 (0.62-2.96)	0.161	1.16 (0.94-1.43)
rs10783299	0.455	0.96 (0.85-1.08)	0.752	0.443	0.95 (0.77-1.15)	0.91 (0.72-1.17)	0.494	0.94 (0.77-1.13)	0.631	0.95 (0.77-1.17)	0.841	0.98 (0.83-1.17)
rs5017427	0.111	0.90 (0.79-1.03)	0.282	0.221	0.89 (0.75-1.07)	0.81 (0.60-1.11)	0.140	0.88 (0.74-1.04)	0.310	0.86 (0.63-1.16)	0.352	0.92 (0.77-1.09)
rs9648724	0.934	1.01 (0.87-1.16)	1.011	0.982	1.01 (0.84-1.20)	1.01 (0.68-1.49)	0.930	1.01 (0.85-1.20)	0.972	1.01 (0.69-1.48)	0.954	1.01 (0.84-1.20)
rs645163	0.521	1.04 (0.92-1.17)	0.791	0.541	1.02 (0.84-1.24)	1.09 (0.85-1.39)	0.670	1.04 (0.87-1.25)	0.521	1.07 (0.86-1.34)	0.921	0.99 (0.84-1.18)
rs6436094	0.842	0.99 (0.88-1.12)	0.75	0.814	0.93 (0.77-1.13)	0.99 (0.78-1.27)	0.583	0.95 (0.79-1.14)	0.773	1.03 (0.83-1.28)	0.450	0.94 (0.79-1.11)
rs6713532	0.983	1.00 (0.88-1.13)	0.664	0.583	0.93 (0.77-1.13)	1.03 (0.79-1.33)	0.631	0.96 (0.80-1.14)	0.564	1.07 (0.85-1.35)	0.373	0.93 (0.78-1.10)
rs16147	0.433	0.95 (0.84-1.08)	0.172	0.092	1.14 (0.95-1.37)	0.79 (0.61-1.04)	0.611	1.05 (0.88-1.24)	0.020	0.74 (0.58-0.96)	0.035	1.20 (1.01-1.43)

Under the codominance model of rs2167270, the OR of genotype GA relative to GG is 1.23, 95% CI is (1.03-1.48), and Pobs = 0.02; under the dominant model, the OR of genotype GA-AA relative to GG is 1.21, 95% CI is (1.02-1.44), and Pobs = 0.029; in the superdominant model, the OR of genotype GA relative to AA-GG is 1.22, 95% CI is (1.02-1.46), and Pobs = 0.028. In the recessive model of rs16147, the OR of genotype TT relative to CC-CT is 0.74, 95% CI is (0.58-0.96), and Pobs = 0.02, relative to CC-CT genotype; TT genotype is a protective factor; under the superdominant model, the OR of genotype CT relative to TT-CC was 1.20, 95% CI was (1.01-1.43), and Pobs = 0.035.

**Table 7:** Robustness test results of the MAX3 method.

Gene	SNP	*χ* ^2^	*P*
LEP	rs4731426	1.839	0.138
	rs2167270	2.184	0.063
LEPR	rs12405556	0.323	0.925
JAK1	rs17127107	1.032	0.533
	rs10889502	1.253	0.389
JAK2	rs7849191	0.129	0.992
STAT3	rs9891119	0.780	0.680
SHP-2	rs4767860	0.914	0.599
PPARGC1A	rs2970847	0.163	0.999
	rs6821591	0.527	0.830
PRKAA1	rs249429	0.975	0.556
	rs3805486	1.078	0.496
PRKAA2	rs1342382	0.647	0.780
	rs6588640	0.867	0.620
PRKAB2	rs6937	1.671	0.191
	rs3766522	1.625	0.186
PRKAG1	rs10783299	0.748	0.710
PRKAG2	rs5017427	1.593	0.226
	rs9648724	0.083	0.997
PRKAG3	rs645163	0.643	0.774
	rs6436094	0.558	0.832
Alpha-MSH	rs6713532	0.589	0.816
NPY	rs16147	2.323	0.045

**Table 8:** Results of adjusting covariates under five genetic models in the LEP pathway.

SNP	Additive		Codominant				Dominant		Recessive		Overdominant	
	*P*	OR (95% CI)	*P*		OR (95% CI)		*P*	OR (95% CI)	*P*	OR (95% CI)	*P*	OR (95% CI)
			1	2	1	2						
rs4731426	0.192	1.09 (0.96-1.25)	0.210	0.171	1.18 (0.98-1.41)	1.14 (0.84-1.54)	0.081	1.17 (0.98-1.39)	0.724	1.05 (0.79-1.41)	0.124	1.15 (0.96-1.37)
rs2167270	0.118	1.12 (0.97-1.29)	0.024	0.110	1.22 (1.01-1.47)	1.12 (0.78-1.63)	0.039	1.20 (1.01-1.44)	0.822	1.04 (0.72-1.50)	0.044	1.21 (1.00-1.45)
rs12405556	0.874	0.99 (0.83-1.17)	0.970	0.920	0.97 (0.80-1.19)	1.01 (0.58-1.76)	0.810	0.98 (0.81-1.18)	0.961	1.01 (0.58-1.76)	0.793	0.97 (0.80-1.19)
rs17127107	0.463	1.05 (0.92-1.20)	0.771	0.484	1.00 (0.84-1.20)	1.12 (0.82-1.53)	0.811	1.02 (0.86-1.22)	0.471	1.12 (0.83-1.51)	0.862	0.98 (0.83-1.17)
rs10889502	0.394	1.06 (0.93-1.21)	0.483	0.406	1.12 (0.93-1.34)	1.11 (0.83-1.48)	0.231	1.11 (0.93-1.33)	0.733	1.05 (0.80-1.38)	0.332	1.09 (0.92-1.30)
rs7849191	0.948	1.00 (0.88-1.13)	0.963	0.910	0.98 (0.81-1.18)	1.01 (0.76-1.33)	0.852	0.98 (0.82-1.18)	0.882	1.02 (0.79-1.32)	0.774	0.97 (0.82-1.16)
rs9891119	0.459	1.05 (0.93-1.19)	0.734	0.580	1.07 (0.87-1.31)	1.10 (0.85-1.41)	0.450	1.08 (0.89-1.30)	0.644	1.05 (0.85-1.31)	0.752	1.03 (0.86-1.22)
rs4767860	0.335	1.07 (0.94-1.21)	0.604	0.511	1.05 (0.87-1.26)	1.16 (0.87-1.55)	0.461	1.07 (0.90-1.27)	0.383	1.13 (0.86-1.49)	0.863	1.02 (0.85-1.21)
rs2970847	0.835	1.02 (0.88-1.17)	0.950	0.893	1.03 (0.85-1.24)	0.99 (0.69-1.42)	0.820	1.02 (0.86-1.22)	0.921	0.98 (0.69-1.40)	0.762	1.03 (0.86-1.23)
rs6821591	0.784	0.98 (0.86-1.12)	0.670	0.634	0.93 (0.77-1.11)	1.02 (0.76-1.38)	0.532	0.95 (0.79-1.13)	0.682	1.06 (0.80-1.41)	0.372	0.92 (0.78-1.10)
rs249429	0.514	1.05 (0.91-1.22)	0.831	0.782	1.05 (0.87-1.27)	1.09 (0.73-1.63)	0.551	1.06 (0.88-1.26)	0.741	1.07 (0.72-1.59)	0.641	1.04 (0.87-1.26)
rs3805486	0.873	0.99 (0.86-1.14)	0.611	0.462	1.06 (0.88-1.27)	0.88 (0.61-1.28)	0.752	1.03 (0.86-1.23)	0.434	0.86 (0.60-1.24)	0.471	1.07 (0.89-1.28)
rs1342382	0.643	0.97 (0.84-1.11)	0.604	0.611	0.92 (0.77-1.11)	1.07 (0.74-1.54)	0.491	0.94 (0.79-1.12)	0.602	1.10 (0.77-1.58)	0.344	0.92 (0.77-1.10)
rs6588640	0.468	0.95 (0.83-1.09)	0.660	0.560	0.94 (0.78-1.13)	0.87 (0.61-1.25)	0.422	0.93 (0.78-1.11)	0.521	0.89 (0.63-1.27)	0.623	0.96 (0.80-1.14)
rs6937	0.208	1.08 (0.96-1.23)	0.421	0.380	1.14 (0.93-1.39)	1.14 (0.88-1.47)	0.190	1.14 (0.94-1.38)	0.684	1.05 (0.84-1.30)	0.390	1.08 (0.91-1.28)
rs3766522	0.209	1.13 (0.93-1.37)	0.481	0.664	1.13 (0.92-1.40)	1.17 (0.53-2.60)	0.232	1.14 (0.92-1.40)	0.752	1.14 (0.51-2.52)	0.250	1.13 (0.91-1.40)
rs10783299	0.479	0.96 (0.85-1.08)	0.790	0.462	0.94 (0.76-1.15)	0.93 (0.72-1.19)	0.492	0.93 (0.77-1.13)	0.753	0.97 (0.78-1.19)	0.722	0.97 (0.81-1.15)
rs5017427	0.121	0.90 (0.79-1.03)	0.314	0.25	0.90 (0.75-1.08)	0.81 (0.59-1.11)	0.172	0.88 (0.74-1.05)	0.292	0.85 (0.62-1.15)	0.434	0.93 (0.78-1.11)
rs9648724	0.953	1.00 (0.87-1.16)	0.971	0.883	1.01 (0.84-1.22)	0.97 (0.65-1.45)	0.921	1.01 (0.85-1.20)	0.864	0.97 (0.65-1.43)	0.863	1.02 (0.85-1.22)
rs645163	0.585	1.04 (0.92-1.17)	0.790	0.552	0.99 (0.81-1.20)	1.07 (0.83-1.38)	0.913	1.01 (0.84-1.22)	0.501	1.08 (0.86-1.35)	0.672	0.96 (0.81-1.15)
rs6436094	0.970	1.00 (0.88-1.13)	0.794	0.830	0.95 (0.78-1.16)	1.01 (0.79-1.30)	0.714	0.97 (0.80-1.16)	0.682	1.05 (0.84-1.30)	0.504	0.94 (0.79-1.12)
rs6713532	0.953	1.00 (0.88-1.14)	0.822	0.713	0.96 (0.79-1.17)	1.04 (0.79-1.35)	0.811	0.98 (0.81-1.18)	0.633	1.06 (0.84-1.34)	0.571	0.95 (0.80-1.13)
rs16147	0.348	0.94 (0.83-1.07)	0.170	0.091	1.13 (0.94-1.37)	0.77 (0.58-1.01)	0.702	1.04 (0.87-1.23)	0.012	0.72 (0.56-0.93)	0.035	1.21 (1.01-1.44)

**Table 9:** Results of haplotype unconditional logistic regression analysis of 8 genes in LD block in the LEP signaling pathway.

Gene	SNP		SNP		Freq	OR (95% CI)	*P*
LEP	rs2167270	G	rs4731426	C	0.695	1.00	—
		A		G	0.238	1.14 (0.99-1.32)	0.076
		G		G	0.067	0.99 (0.77-1.27)	0.921

JAK1	rs10889502	C	rs17127107	C	0.352	1.00	—
		G		C	0.333	1.09 (0.94-1.27)	0.261
		C		G	0.304	1.07 (0.91-1.25)	0.411
		G		G	0.011	1.57 (0.74-3.37)	0.240

PPARGC1A	rs2970847	C	rs6821591	T	0.673	1.00	—
		T		C	0.238	1.00 (0.86-1.15)	0.950
		C		C	0.087	0.95 (0.76-1.19)	0.674

PRKAA1	rs249429	T	rs3805486	A	0.546	1.00	—
		T		G	0.234	1.01 (0.87-1.18)	0.881
		C		A	0.221	1.05 (0.90-1.23)	0.532

PRKAA2	rs1342382	T	rs6588640	G	0.496	1.00	—
		A		G	0.253	0.95 (0.82-1.11)	0.531
		T		A	0.250	0.93 (0.80-1.07)	0.300

PRKAB2	rs3766522	A	rs6937	C	0.541	1.00	—
		A		T	0.340	1.05 (0.92-1.21)	0.481
		T		T	0.118	1.14 (0.93-1.39)	0.212

PRKAG2	rs2970847	C	rs6821591	T	0.673	1.00	—
		T		C	0.238	1.00 (0.86-1.15)	0.951
		C		C	0.087	0.95 (0.76-1.19)	0.673

PRKAG3	rs6436094	A	rs645163	C	0.433	1.00	—
		G		T	0.422	1.02 (0.89-1.16)	0.801
		G		C	0.137	0.95 (0.79-1.15)	0.621

**Table 10:** Significant results of SNP-SNP interaction of genes in the LEP signaling pathway.

CHR1	SNP1	CHR2	SNP2	OR_INT∗	*χ* ^2^	*P*	Genes
7	rs16147	12	rs1044471	1.311	8.390	0.004	NPY×ADIPOR2
7	rs2167270	17	rs5435	1.422	9.870	0.002	LEP×SLC2A4

**Table 11:** SNP set analysis results based on the LEP pathway.

Model	Kernel	*Q*	*P*	Resampling *P*
Without covariates	Linear	2072.896	0.673	0.672
	Linear.weighted	211.471	0.116	0.115
	IBS	73.692	0.684	0.686
	IBS.weighted	103.078	0.134	0.124

With covariates	Linear	2132.481	0.631	0.641
	Linear.weighted	211.693	0.114	0.120
	IBS	77.226	0.603	0.620
	IBS.weighted	104.682	0.128	0.129

## Data Availability

The data used to support the findings of this study are available from the corresponding author upon request.
